# Don't-Ask-Don't-Tell Policy Strangles Research and Do-Gooder Intentions Ablaze

**DOI:** 10.1100/tsw.2001.54

**Published:** 2001-06-01

**Authors:** Shauna M. Haley

## Abstract

Nature starts the news week by chronicling epidemiological researchers' wrath over how Britain's General Medical Council (GMC) has implemented the nation's 1998 Data Protection Act. Science fires up its news with coverage of the torching of two plant labs in the northwestern U.S. by ecoterrorists.

Amidst rising worldwide concern about the potential backlash on medical research capabilities of electronic-age data protection laws, Britain's major medical regulatory agency is receiving sharp comments from the nation's leading epidemiologists, reports *Nature*. The row revolves around the GMC's translation of a 1998 law that forbids personal information from being used in any manner other than one for which informed consent is granted. Prior to the Data Protection Act, physicians in Britain shared patient data with epidemiological researchers under a certain portion of the GMC's guidelines. However, the agency issued new guidelines designed to enforce the Data Protection Act last September, informing physicians that this sharing of patient information would henceforth be considered illegal, unless informed consent is granted by each patient. This new move has put a chokehold on the medical research community, who can no longer even determine where study-eligible patients are being treated, says Institute of Cancer Research Epidemiology head Julian Peto in *Nature*. A GMC spokesperson insists that the agency has handled the law appropriately, but says in *Nature* that consumer and physician groups will band together to review and advise the Secretary of State for Health on specific projects. The Secretary of State for Health has the power to override the consent rules set down by the GMC. Researchers in Britain say the move might not be enough to save some pending research projects.

The University of Washington (UW) in Seattle and a private facility in Portland, Ore., are the latest victims in a year-long wave of vandalism against plant genetics researchers, says *Science*. Both research facilities, however, used traditional — not biotech — methods to improve the trees they studied. The group Earth Liberation Front (ELF) is suspected to have set both fires, but had not claimed responsibility for either arson at TWINS press time. A recent addition to the ELF site, though, is an instruction manual entitled, "Setting Fires with Electrical Timers." (TWINS must ask: If one has the intellectual sophistication to build an electrical timer to start a fire, then is that one also not able to figure out that destroying endangered plants will actually prevent the restoration of 'the wild'?) The UW fire was ominously personal and caused the loss of critical pieces of research aimed at restoring endangered plants and wetlands, notes *Science*. Professor Toby Bradshaw, whose lab appears to have been the primary target of the arsonist(s), told the journal that the perpetrator had to have known the researcher's work habits— two boxes usually containing pet snakes used in lectures to demonstrate inheritance had been moved out of the range of the fire. ELF had previously criticized Bradshaw because part of his grant funding came from commercial tree companies, who might benefit from genetic study results. (Far be it for a corporation to make an investment in science with the possibility of a return.) The attack on the UW facility marks an escalation of ecoviolence into the realm of non-commercial research that has not previously been commonplace. This new direction for domestic terrorism could prove worrisome for the agricultural sciences.

This Week in Nature and Science, June 1, 2001
© 2001 TheScientificWorld, Inc.
